# Monitoring and evaluation of health disparities for people with disability in low- and middle-income countries: a scoping review

**DOI:** 10.1093/epirev/mxag009

**Published:** 2026-03-24

**Authors:** Sarah Walmsley, Marie Huska, Zoe Aitken, Manjula Marella, Anne Kavanagh, Kaloyan Kamenov, Darryl Barrett, Alexandra Devine

**Affiliations:** Centre for Health Policy, Melbourne School of Population & Global Health, University of Melbourne, Melbourne, Australia; Centre for Health Policy, Melbourne School of Population & Global Health, University of Melbourne, Melbourne, Australia; Centre for Health Policy, Melbourne School of Population & Global Health, University of Melbourne, Melbourne, Australia; Nossal Institute for Global Health, Melbourne School of Population & Global Health, University of Melbourne, Melbourne, Australia; Centre for Health Policy, Melbourne School of Population & Global Health, University of Melbourne, Melbourne, Australia; Disability Programme, Department for Noncommunicable Diseases and Mental Health, World Health Organization, Geneva, Switzerland; Disability Programme, Department for Noncommunicable Diseases and Mental Health, World Health Organization, Geneva, Switzerland; Centre for Health Policy, Melbourne School of Population & Global Health, University of Melbourne, Melbourne, Australia

**Keywords:** persons with disability, health equity, developing countries, epidemiologic studies

## Abstract

People with disability experience health inequities and mostly live in low- and middle-income countries (LMICs), because that is where most of the world’s population resides. Despite this, existing evidence on health equity for people with disability mostly comes from high-income settings. Monitoring and evaluation of health equity are crucial for countries to address and track progress toward goals, such as the highest attainable standard of health for people with disability. This scoping review summarizes the available literature on approaches and indicators used in LMICs to evaluate health-related outcomes between people with and without disability. Peer-reviewed articles were included that were published between 2008 and 2024 that compared health-related outcomes between these 2 populations. We identified 59 eligible studies from a broad range of LMICs. Disability indicators varied, with most studies using 1 of multiple Washington Group question sets to enable disaggregation of data by disability status. Survey data were the type most frequently used; only 2 studies used administrative data. A wide range of health-related outcomes were explored; themes of maternal and child health and HIV-related outcomes emerged as key areas of focus. Disparities were consistently found, with almost all included studies reporting poorer outcomes for people with compared with people without disability. There was a noticeable lack of action taken to improve future policy or monitoring and evaluation or to enact real and meaningful change in health equity for people with disability.

## Introduction

The United Nations Convention on the Rights of Persons with Disabilities (UNCRPD), enacted in 2008, asserts the right of people with disability to the highest attainable standard of health (Article 25).^1^ In 2022, the World Health Organization (WHO) launched the Global Report on Health Equity for People with Disabilities in response to the resolution (WHA74.8) reaffirming the need for countries to ensure people with disability can “exercise their full right to health.”^2^^,^^3^ The report highlights significant health inequities (related to morbidity, mortality, and functioning) for people with disability.^3^ Health inequities for people with disability are unfair and unjust disparities in mortality, morbidity, and functioning which cannot be explained by underlying health conditions.^3^ These inequities stem from social, economic, and environmental determinants of health such as poverty, poor housing conditions, or poor access to health care, which disproportionately burden people with disability.^3^^,^^4^ Health inequalities encompass both health inequities and unavoidable differences in health and health outcomes, which may be “explained to some extent by underlying health conditions” associated with disability.^4^ Distinguishing whether studies measure inequities or inequalities can be challenging, so for the purpose of this review, we use the term *disparities* to encompass both concepts in relation to health outcomes.

Much of the available evidence presented in the Global Report draws on data from high-income countries (HICs).^3^ Even though, nearly 80% of the world’s 1.3 billion people with disability live in low- and middle-income countries (LMICs). Health disparities for people with disability may be exacerbated in LMICs due to barriers in these settings, including lack of financial health system resourcing. For example, in a global scoping review, Gréaux et al.^5^ identified greater reporting of financial barriers to health care access for people with disability in LMICs compared with those residing in HICs. The mortality rate is higher for people with disability globally; however, in a recent review, Kuper et al.^6^ found a greater gap in life expectancy in LMICs than HICs. Despite recognition that disparities may be amplified for people with disability in low-resource settings, both reviews highlighted the limited availability of evidence from LMICs.

The Global Report outlined 10 strategic entry points in the health system to advance health equity for people with disability.^3^ The report recognized the importance of monitoring and evaluation (strategic point 9), which aids countries in evaluating existing gaps in health equity for people with disability, to establish priorities and set baselines for improvement. The analysis of disability data is essential to guide the development of evidence-based policy and disability-inclusive action plans. These methods also enable countries to monitor progress toward the Sustainable Development Goals (SDGs) of the 2030 Agenda for Sustainable Development^7^ and the UNCRPD.^1^ Article 31 of the UNCRPD asserts the legal obligations of signatories to monitor the progress of implemented policies through the collection of relevant data.^1^ Because the UNCRPD is approaching complete ratification, many countries have embarked on collecting some form of disability-related data.

To compare health-related outcomes between people with and without disability, countries need data sources that contain a measure of disability status (ie, a disability indicator that allows for data to be disaggregated by disability). Health outcome data are also required, which could include a range of measures related to morbidity, mortality, and access to health care services.^3^

The aim of this scoping review was to summarize the current approaches used to monitor and evaluate health-related disparities between people with and without disability in LMICs. We aimed to understand the methods adopted in LMICs to monitor and evaluate health disparities between people with and without disability, with a focus on disability indicators used to disaggregate data, data sources used, and health-related outcomes measured (including morbidity, mortality, knowledge, attitudes, practice, and access to health care services).

## Methods

The scoping review was conducted in accordance with the Levac et al.^8^ revision of the Arksey and O’Malley methodological framework.^9^ The framework consists of a 5-stage process: identifying the research question; identifying relevant studies; study selection; charting the data; and collating, summarizing, and reporting results. The protocol for this review was registered on OSF (https://doi.org/10.17605/OSF.IO/ZT3JM).

### Research question

Our key research question was: What approaches have been adopted in LMICs to disaggregate disability data and compare health-related outcomes between people with and without disability?

### Search strategy

Using the MEDLINE, SCOPUS, and Global Health databases, we systematically searched on July 22, 2024, the literature for relevant studies published between 2008 and 2024. After full-text screening, the reference lists of all included studies were searched for additional studies to include. The Cochrane Effective Practice and Organization of Care LMIC filter (2023 version)^10^ was adapted and combined with terms related to disability, health-related outcomes, and data sources. The search strategy was developed in consultation with an expert librarian (Table S1).

### Selection criteria

Here we outline study selection criteria in line with the JBI Manual for Evidence Synthesis framework of population, concept, context, and types of sources.^11^

#### Population

We included peer-reviewed publications that identified and contextualized the population of interest as people with disability and included a comparison population of people without disability.

#### Concept

We included any study that compared health-related outcomes between people with and without disability in LMICs, using population-based survey data, Health and Demographic Surveillance Systems, or administrative data (data collected during the day to day running of services, such as civil registration and vital statistics systems and health information systems [HIS]). Health-related outcomes were broadly defined as morbidity, mortality, knowledge/attitudes/practice, and/or health care access. Studies from HICs or that did not use data from relevant sources were excluded.

#### Context

The context of this review was LMICs, based on the World Bank classification of countries for the 2024 financial year, defined as a gross national income per capita of US$13 845 or less.^12^ Classification of LMICs was based on the location of the data used in the study (ie, the study setting). The focus of this review was the methods and approaches being implemented to better understand what is possible within LMIC contexts, which are often resource limited.

#### Types of sources

We included quantitative primary research studies published between May 2008 and July 2024. Qualitative studies, reviews, and conference abstracts were not included.

### Study selection

**Figure 1:**
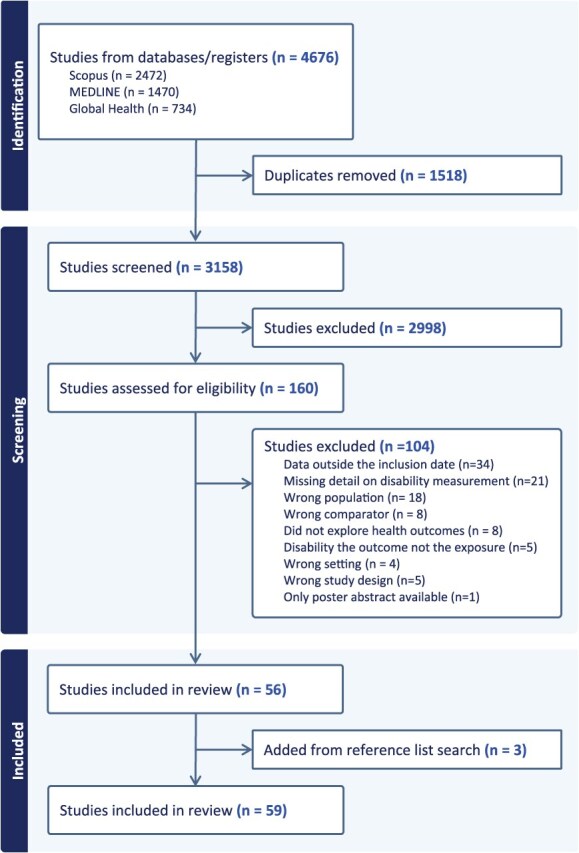
Flow chart of study selection.

A total of 4676 studies were imported into Covidence software for title and abstract screening. After removal of duplicates, the titles and abstracts of 3158 studies were screened against inclusion criteria (Figure 1). Of these, 160 studies were retained for full-text screening, after which 104 studies that did not meet the study criteria were excluded. The most common reasons for exclusion at this stage were that the study included data collected before 2008 or that the article was missing details on how disability was measured. The reference lists of the 56 included studies were searched, leading to an additional 3 studies being identified for inclusion. Three reviewers (S.W., A.D., and M.H.) screened the articles, with each article being reviewed by 2 authors at every step.

### Data extraction

Data were extracted from the first 15 studies by 1 reviewer (S.W.) using an extraction form in Covidence developed by the authors. Following discussion, the extraction form was then updated with additional items before extraction was completed by 1 reviewer on all articles. The following data were extracted from included studies: year, country or countries, region(s), primary aim, number of participants, population characteristics (eg, sex, age, disability type), study design, data type, data source, data collection methods for primary research, data analysis method, disability indicator, health-related outcomes measured, and relevant findings.

### Data synthesis

We first organized the data in tabular format sorted by region and country and used a descriptive numeric approach to summarize the main characteristics of the included studies. This enabled comparisons across studies and regions. We then used a descriptive analytical approach to thematically summarize findings relevant to the review questions, based on a predefined framework. The framework was developed by the research team, and analysis was conducted by 1 reviewer (S.W.), with another (A.D.) overseeing the process.

## Results

### Study characteristics

In total, 59 studies were identified that met the inclusion criteria for this review. Study characteristics are outlined in detail in Table S2. Articles reporting on the studies were published between 2010 and 2024; half of the articles were published within the past 4 years.^13‑41^ Of the 59 studies, 5 were conducted in East Asia and the Pacific,^22^^,^^35^^,^^41‑43^ 12 in Latin America,^14^^,^^19^^,^^20^^,^^29^^,^^33^^,^^36^^,^^44‑49^ 1 in the Middle East and North Africa,^50^ 13 in South Asia,^13^^,^^17^^,^^24^^,^^25^^,^^27^^,^^28^^,^^30^^,^^32^^,^^51‑55^ and 22 in Sub Saharan Africa.^15^^,^^18^^,^^23^^,^^26^^,^^31^^,^^34^^,^^37^^,^^38^^,^^56‑69^ The remaining 6 studies included data from multiple regions.^16^^,^^21^^,^^39^^,^^40^^,^^70^^,^^71^

The characteristics of populations included in the studies varied. Seven studies included children only (aged <18 years),^19^^,^^34^^,^^37^^,^^40^^,^^47^^,^^59^^,^^60^ 14 included adults only (aged ≥18 years),^15^^,^^16^^,^^20^^,^^33^^,^^42^^,^^43^^,^^49^^,^^51^^,^^52^^,^^61‑64^^,^^70^ 32 included children and adults,^13^^,^^14^^,^^17^^,^^18^^,^^22‑27^^,^^29‑32^^,^^36^^,^^38^^,^^39^^,^^45^^,^^46^^,^^50^^,^^53‑58^^,^^65‑69^^,^^71^ and 6 looked at populations of older adults (aged ≥60 years).^21^^,^^28^^,^^35^^,^^41^^,^^44^^,^^48^ Ten studies only included female participants; several studies further limited inclusion based on marital status or recent pregnancy.^17^^,^^18^^,^^23^^,^^25‑27^^,^^30^^,^^32^^,^^54^^,^^55^ No studies focused solely on male participants. Three studies looked at sub-populations of people with HIV-positive status,^31^^,^^59^^,^^64^ and 1 study looked at a sub-population of Venezuelan refugees in Peru.^33^

### Design of studies and data sources

Thirty-six of the 59 studies used a cross-sectional design.^16^^,^^18‑20^^,^^23^^,^^25‑34^^,^^36‑41^^,^^43^^,^^44^^,^^47‑49^^,^^55^^,^^56^^,^^60^^,^^62‑65^^,^^68‑70^ Of the remaining studies, 11 were case-control studies,^17^^,^^50‑52^^,^^54^^,^^57‑59^^,^^61^^,^^66^^,^^67^ 7 were nested case-control studies,^13^^,^^21^^,^^24^^,^^45^^,^^46^^,^^53^^,^^71^ 4 were cohort studies,^14^^,^^15^^,^^35^^,^^42^ and 1 used mixed-methods.^22^ Only 2 studies used administrative data; 1 used benefit payment and date of death data from the country’s National Social Security Administration database,^14^ and another linked survey data with data from electronic medical records.^59^ Thirty-two studies conducted secondary analysis of existing data from surveys, Health and Demographic Surveillance Systems, or longitudinal studies.^15^^,^^16^^,^^18‑21^^,^^23^^,^^25^^,^^27‑42^^,^^44^^,^^47^^,^^48^^,^^56^^,^^62^^,^^63^^,^^65^^,^^69^ Surveys with disability data available included annual and national health surveys, demographic and health surveys, and household surveys including the multiple indicator cluster survey (MICS). Topic-specific surveys were used in some studies, including those focused on HIV, aging, violence against children, and migrant populations. Only 4 studies included longitudinal data or repeated measures over time,^14^^,^^15^^,^^35^^,^^42^ with the remaining studies assessing disparities at a single point in time.

Twenty-six of the 59 studies conducted primary research to assess disparities in health-related outcomes.^13^^,^^17^^,^^22^^,^^24^^,^^26^^,^^43^^,^^45^^,^^46^^,^^49‑55^^,^^57‑61^^,^^64^^,^^66‑68^^,^^70^^,^^71^ Several of these studies reported on the methods and resources used for data collection. Seventeen studies reported that questionnaires and surveys were translated into local languages,^13^^,^^22^^,^^26^^,^^43^^,^^45^^,^^46^^,^^49^^,^^50^^,^^52‑55^^,^^60^^,^^66^^,^^67^^,^^70^^,^^71^ and 17 field- or pilot-tested questionnaires.^13^^,^^17^^,^^22^^,^^26^^,^^43^^,^^46^^,^^49^^,^^50^^,^^52‑55^^,^^58^^,^^60^^,^^61^^,^^70^^,^^71^ Eight studies described consultation with people with disability, and organizations of persons with disabilities (OPDs) during the study design and development of survey tools.^43^^,^^46^^,^^50^^,^^52^^,^^58^^,^^61^^,^^70^^,^^71^ Two studies used pictorial guides to train collectors on the identification of people with disability.^52^^,^^54^ Five studies specifically included people with disability in the data collection team.^43^^,^^51‑53^^,^^70^ Several studies also described the incorporation of accessible material in the data collection methodology. One study provided a simple language statement,^51^ interviews using sign language were conducted in 2 studies,^17^^,^^55^ and another study provided pictorial aids to illustrate questions.^60^

### Disability indicators based on functioning limitations

The disability indicators used in the included studies are summarized in Table 1 (detailed information available is provided in Table S2). Fifty-seven of the 59 studies focused on more than 1 type of disability. Fifty-three studies used disability indicators relying on functioning limitations; of those studies, 33 used Washington Group (WG) question sets to identify disability, with the Short Set being used most frequently (*n* = 22 studies). Thirty-one of these studies used WG questions alone, and 2 used WG questions in combination with the identification of people through registration with the National Social Protection.^13^^,^^24^ The WG response cutoffs to disaggregate by disability status varied, with a general trend that inclusion of “some difficulty” in the categorization of disability led to increased prevalence estimates. This was evident in studies that applied multiple cutoffs. For example, Mahmood et al.^27^ defined disability as reporting some difficulty or more in at least 1 functional domain, and severe disability as a response of “a lot of difficulty” or more in at least 1 domain. Using the lower cutoff (ie, some difficulty), the estimated prevalence of disability was 14.1%, whereas using the higher cutoff resulted in a prevalence of 2.6% (severe disability). Some studies used the WG questions to binarily categorize functioning difficulties (albeit in varied ways); however, others used them to disaggregate by severity of functioning difficulties, either categorically or using summed scores.

**Table 1:** Summary of disability indicators used in included studies.

**Disability indicator**	**First author, year**
ADL scales^a^	Muhammad, 2022^28^; Yan, 2023^35^
Assessment by key informant	Gudlavalleti, 2014^35^; Murthy, 2014^54^
Disability Screening Questionnaire-34	Trani, 2018^50^
ICF-based 35-item screening tool	Trani, 2011^61^
Multiple tools used to assess intellectual disability	Aderemi, 2013^60^
Identification through OPD lists	Carew, 2019^58^
Katz Index of Independence in ADL	Flores-Flores, 2018^48^; He, 2019^42^
Multiple questions^a^	Barreto, 2023^29^; Lopez-Gil, 2021^19^; Macarevich Condessa, 2021^20^; Pengpid, 2019^65^; Shi, 2024^41^; Silva, 2017^44^; Yan, 2023^35^
Rapid Assessment of Disability survey	Grills, 2017^51^; Marella, 2014^70^; Marella, 2016^43^; Mathias, 2018^53^^b^
Receipt of disability-related payment	Banks, 2020^13^; Banks, 2022^24^; Grushka, 2020^14^
Registration with social protection	Banks, 2020^13^
Self-reported health condition	Banks, 2022^24^
Single question	Casebolt, 2022^25^; Casebolt, 2023^30^; Muhammad, 2022^28^; Rohrer, 2010^49^
WG (set not specified)	Devkota, 2021^17^; Hernandez-Vasquez, 2023^33^
WG Extended Set	Banks, 2020^13^; Banks, 2022^24^; DeBeaudrap, 2019^57^; Kuper, 2018^45^; Mactaggart, 2016^71^; Prynn, 2021^21^; Wallace, 2020^16^^b^
WG Short Set	Abimanyi-Ochom, 2017^69^; Carew, 2019^58^^c^; Casebolt 2024^36^; Chipanta, 2022^26^; Chipanta, 2023^31^; Danquah, 2015^46^; Devkota, 2017^55^; Eide, 2015^68^; Hameed, 2023^32^; Kwagala, 2021^18^; Mahmood, 2022^27^; Massetti, 2024^38^^b^; Moodley, 2015^62^; Mutwali, 2019^63^; Onadja, 2013^56^; Prynn, 2020^15^; Prynn, 2021^21^; Rotenberg, 2024^39^; Vergunst, 2017^67^; Vergunst, 2019^66^; Wilbur, 2021^22^; Zandam, 2021^23^
WG/UNICEF CFM	Banks, 2020^13^; Banks, 2022^24^; de Castro, 2017^47^; Ekman, 2024^37^; Kuper, 2018^45^; Mactaggart, 2016^71^; Rotenberg, 2023^34^; Rotenberg, 2024^39^; Rotenberg, 2024^40^
WHO Disability Assessment Schedule 2.0	Myezwa, 2016^64^
WHO Ten Question Screen	Devendra, 2013^59^

Abbreviations: ADL, activities of daily living; CFM, Child Functioning Module; ICF, International Classification of Functioning, Disability, and Health; OPD, organizations of persons with disabilities; UNICEF, United Nations Children’s Fund; WG, Washington Group; WHO, World Health Organization.

a The tool was not specified.

b A modified version of the tool was used.

c Used for sensitivity analysis only.

Of the remaining 20 studies that used functioning limitation, disability indicators included the Disability Screening Questionnaire-34,^50^ the Rapid Assessment of Disability,^43^^,^^51^^,^^53^^,^^70^ the Katz Index of Independence in Activities of Daily Living,^42^^,^^48^ the WHO Disability Assessment Schedule,^64^ and the WHO Ten Question Screen.^59^ One study asked a single question: “Are you limited in any way in any activities because of physical, mental or emotional problems?”^49^ One study that only focused on intellectual disability used Raven’s Progressive Matrices, the Draw-A-Person Test for learners, and the Vineland’s Social Maturity Scale for caregivers of learners.^60^ Another focused on psychosocial disability used a modified Kessler-6 scale as part of the Rapid Assessment of Disability survey.^53^

Indicators based on functional limitations generally measured functioning with the use of assistive devices (eg, glasses). Studies that explored the availability of assistive devices found high levels of unmet need. For example, Mactaggart et al.^71^ found that only 48% of participants who needed assistive devices and services had received access.

### Other disability indicators

Six studies did not use measures of functioning difficulty as their disability indicator. Two of these 6 studies used a multistage process to measure disability, with the initial listing based on key-informant assessment (visible assessment and taking a short medical history) supported by information from disability certificates and disability support pension records. The listing was then checked and confirmed by medically trained physicians or therapists.^52^^,^^54^ Another study used lists of people with disability collated by an OPD, with a comparison group of age- and sex-matched control participants without disability.^58^ Whereas the primary analysis relied on the OPD determination of disability, the study team cross-checked their findings using WG Short Set.^58^ Two studies asked women if they had any disability type.^25^^,^^30^ The final study based disability identification on receipt of disability pension payments, eligibility for which was determined by the impact of disability on individuals’ ability to work.^14^

Although comparing disability prevalence was not a primary aim of this review, we did extract prevalence estimates (available in Table S3). There was considerable heterogeneity across studies in terms of the demographic characteristics of populations and sub-regions included; therefore, prevalence estimates may not be comparable or reflective of disability prevalence across regions.

### Data analysis

Various statistical methods were used to compare outcomes between people with and without disability. A majority of studies expressed differences in the multiplicative scale,: 42 the studies presented odds ratios, primarily calculated using logistic regression,^13^^,^^16‑26^^,^^28‑32^^,^^34‑38^^,^^42‑46^^,^^49‑54^^,^^56‑59^^,^^61^^,^^64^^,^^65^^,^^69^^,^^71^ and 5 studies used Poisson regression to obtain risk or prevalence ratios.^15^^,^^33^^,^^39^^,^^40^^,^^48^ Linear regression was used to compare the difference in outcomes between groups in 5 studies.^41^^,^^55^^,^^58^^,^^66^^,^^67^

### Health-related outcomes measured

A wide range of health-related outcomes were explored. The outcomes broadly included morbidity, mortality, access to health care and community, and knowledge/attitudes/practice (Table 2). Disparities for people with disability were found in a majority of studies, with some studies finding that outcomes varied by disability type and severity. These results indicate widespread disparity across multiple domains of health and health care for people with disability. The outcomes of included studies are outlined in detail in Table S2.

**Table 2:** Summary of health-related outcomes explored in included studies.

**Health-related outcome category**	**First author, year**
Access	Banks, 2020^13^; Banks, 2022^24^; Casebolt, 2023^30^; Chipanta, 2023^31^; Danquah, 2015^46^; de Castro, 2017^47^; DeBeaudrap, 2019^57^; Devendra, 2013^59^; Devkota, 2017^55^; Devkota, 2021^17^; Eide, 2015^68^; Ekman, 2024^37^; Flores-Flores, 2018^48^; Grills, 2017^51^; Gudlavalleti, 2014^52^; Hameed, 2023^32^; Kuper, 2018^45^; Macarevich Condessa, 2021^20^; Mahmood, 2022^27^; Marella, 2014^70^; Marella, 2016^43^; Mathias, 2018^53^; Moodley, 2015^62^; Murthy, 2014^54^; Mutwali, 2019^63^; Prynn, 2021^21^; Rotenberg, 2023^34^; Shi, 2024^41^; Silva, 2017^44^; Trani, 2011^61^; Trani, 2018^50^; Vergunst, 2017^67^; Zandam, 2021^23^
Knowledge, attitudes, and practice	Abimanyi-Ochom, 2017^69^; Aderemi, 2013^60^; Casebolt, 2022^25^; Chipanta, 2022^26^; Chipanta, 2023^31^; Danquah, 2015^46^; DeBeaudrap, 2019^57^; Devkota, 2017^55^; Lopez-Gil, 2021^19^; Mactaggart, 2016^71^; Massetti, 2024^38^; Mutwali, 2019^63^; Myezwa, 2016^64^; Pengpid, 2019^65^; Prynn, 2021^21^; Rotenberg, 2024^39^; Shi, 2024^41^; Trani, 2011^61^; Vergunst, 2019^66^; Zandam, 2021^23^
Morbidity	Banks, 2020^13^; Banks, 2022^24^; Barreto, 2023^29^; Casebolt 2024^36^; Chipanta, 2023^31^; de Castro, 2017^47^; Devendra, 2013^59^; Ekman, 2024^37^; Gudlavalleti, 2014^52^; He, 2019^42^; Kuper, 2018^45^; Mactaggart, 2016^71^; Massetti, 2024^38^; Moodley, 2015^62^; Muhammad, 2022^28^; Murthy, 2014^54^; Myezwa, 2016^64^; Pengpid, 2019^65^; Prynn, 2021^21^; Rotenberg, 2023^34^; Rotenberg, 2024^40^; Vergunst, 2019^66^; Wallace, 2020^16^; Wilbur, 2021^22^; Yan, 2023^35^
Mortality	Grushka, 2020^14^; Prynn, 2020^15^
Other	
Well-being	Carew, 2019^58^; Grills, 2017^51^; Marella, 2014^70^; Marella, 2016^43^
Unmet food needs	Hernandez-Vasquez, 2023^33^
Early childbearing	Kwagala, 2021^18^
Sexual violence	Massetti, 2024^38^
Self-rated health	Moodley, 2015^62^; Onadja, 2013^56^; Rohrer, 2010^49^; Trani, 2011^61^

Two studies compared mortality rates between people with and without disability. Grushka et al.^14^ measured all-cause mortality, finding that the relative risk of mortality was twice as high for people with disability compared with the country-standardized mortality rate in Argentina. Prynn et al.^15^ found higher risk of all-cause and noncommunicable disease-related mortality for people with disability, and that associations varied by impairment type in Malawi.

Twenty-five studies included morbidity as an outcome. The morbidities explored in the studies included chronic illness (eg, diabetes, hypertension, arthritis),^21^^,^^24^^,^^29^^,^^45^^,^^52^^,^^54^^,^^62^^,^^71^ mental health,^16^^,^^35^^,^^42^^,^^45^^,^^54^^,^^64^^,^^66^ communicable diseases (eg, HIV, common childhood illnesses),^31^^,^^34^^,^^36-38^^,^^59^^,^^62^^,^^65^ malnutrition,^40^^,^^47^ cognitive and vision impairments,^28^ incontinence,^22^ and general measures of health.^13^^,^^24^^,^^66^

Other health-related outcomes explored in the articles included access to various types of health care, health-promoting or health risk behavior, and knowledge/attitudes/practice. Some key outcomes were common across several studies, with 10 studies specifically focused on maternal and pregnancy care access,^17^^,^^18^^,^^26^^,^^27^^,^^30^^,^^32^^,^^54^^,^^55^^,^^57^^,^^61^ and 13 focused on HIV and sexual and reproductive health issues (ie, access to health care and testing and knowledge/behaviors).^23^^,^^25-27^^,^^31^^,^^38^^,^^39^^,^^57^^,^^59‑61^^,^^65^^,^^69^ Of 11 studies that explored health-related outcomes in cohorts of women of childbearing age, 10 focused on issues related to antenatal, postnatal, and sexual and reproductive care access.^17^^,^^26^^,^^27^^,^^30^^,^^32^^,^^54^^,^^55^^,^^57^^,^^61^ In study cohorts of children aged 2 to 4 years, authors explored health care–seeking behavior of caregivers for common childhood illnesses.^34^^,^^40^ They found higher rates of illness for children with disability, but similar levels of health care seeking by their parents compared with parents of children without disability. HIV-related studies revealed evident gaps in knowledge of HIV transmission,^39^^,^^60^^,^^69^ lower contraception use,^25^^,^^60^ and higher rates of sexually transmitted disease and HIV^31^^,^^38^^,^^69^ for people with disability compared with those without disability.

Seven of the studies found positive outcomes for people with disability in some of the outcomes explored.^26^^,^^29^^,^^32^^,^^34^^,^^41^^,^^48^^,^^69^ For example, Chipanta et al.^26^ found a higher odds of HIV testing for women with mild or moderate cognitive disability. Other positive outcomes included higher likelihood of receiving blood pressure testing in the previous 2 years for older adults with disability,^48^ greater receipt of components of antenatal care and advice including on exclusive breastfeeding and balanced diet for women with or at risk of disability,^32^ greater likelihood of receiving home health services for older adults with mobility and cognitive disability,^41^ and lower levels of smoking and alcohol consumption for individuals with intellectual, physical, and multiple impairments.^29^ Only 1 of the included studies discussed actions taken to inform policy or enact change as a result of the findings.^43^

## Discussion

In this scoping review, we aimed to map the approaches being adopted to monitor and evaluate health disparities between people with and without disability in LMICs. We found that the approaches used to identify people with disability and to evaluate the wide range of health-related outcomes disaggregated by disability status were varied.

### Summary of evidence

More than half of the included studies were published between 2020 and 2024. This suggests an increasing interest in measuring and reporting on health disparities for people with disability. The included studies used data from a wide range of LMICs from different regions, indicating that there is broad interest in understanding health disparities for people with disability globally, through the examination of health-related outcomes in these settings. There was a noticeable lack of evidence from some regions; for instance, we only found 1 study from the Middle East and North Africa.^50^

Quantitative methods assist in describing the health status of population subgroups, measuring the magnitude of health disparities, and tracking progress toward local, national, and international equity goals.^1^^,^^3^^,^^7^ Various sources of population-based data can be drawn upon for monitoring and evaluation approaches.^72^ We found that survey data were most commonly used, with studies often leveraging existing data from large national or multinational surveys such as demographic and health surveys,^15^^,^^18^^,^^23^^,^^27^^,^^32^^,^^36^^,^^56^^,^^69^ and household surveys including MICS,^34^^,^^37^^,^^39^^,^^40^ which include measures of disability in the standard data collection.

Administrative data (eg, HIS, civil registration, vital statistics systems) are a potential source that can be used to monitor health-related disparities for people with disability. In this scoping review, we found that few studies used data from administrative sources, such as social security^14^ and medical records data.^59^ The WHO Disability-Inclusive Health Services Toolkit^73^ recommends that disability indicators be included in HIS data to monitor health needs and service usage for people with disability. Pilot studies in several LMICs, including Paraguay, Cambodia, Tanzania, and Bangladesh, have collected WG data in hospital and community-based settings to assess accessibility needs of health service users.^74‑77^ Although these studies demonstrate the utility of disability measures in these contexts and present opportunity for future evaluation of health-related outcomes disaggregated by disability status, they have not yet been used for this purpose.

Administrative data could provide benefits for monitoring of health equity, offering the potential to overcome limitations of commonly used sources such as population-based surveys. Surveys can be expensive to implement, often lack granularity at the local administrative division level, and only provide data from a single point in time.^78‑80^ Administrative data, however, are routinely collected in a continuous way, adding little to no additional burden for collection and providing timely longitudinal data that can be used to establish baselines and monitor outcomes and progress.^79^ Concerns about the quality, completeness, accuracy, and timeliness of administrative data in LMICs may impede use of these sources for research purposes.^78^^,^^79^ Additionally, in the absence of appropriate policies for the sharing of administrative data, survey data may be more readily available for secondary analysis, leading researchers to favor more easily accessible sources.^78^ Due to these challenges, population-based surveys are still considered the gold standard for research. Health information systems have been implemented in many LMICs, and in light of recent progress away from paper-based systems,^78^ the use of administrative data, including linking administrative data across systems, could improve future capacity over time for countries to monitor and evaluate health outcomes for people with disability. Interventions that improve data availability, infrastructure, and planning, and build capacity through training could further improve the quality of administrative data available.^81^^,^^82^

Recently, some HICs have used data linkage methods combining data from multiple sources, including administrative data, to identify populations of people with disability and evaluate health-related outcomes.^83‑85^ In this scoping review, only 1 study linked population-based survey data to medical records data for this purpose.^59^ Barriers to the use of these methods in LMICs are multifaceted, reflecting high-level issues such as the need to develop and implement policy for data collection and use, the need to establish HIS architecture, possible transition from paper-based to computer-based systems, and management of strain on systems that are often limited in resources and capacity.^86^ Other factors may include data-related issues, such as the absence of appropriate disability data collection within systems or lack of appropriate processes and governance for the sharing of data across systems and sectors.^86^

Most of the included studies used definitions of disability aligned with the International Classification of Functioning, Disability and Health (ICF), a widely accepted framework for defining disability, based on the biopsychosocial model of disability.^87^ This model recognizes disability as the dynamic interaction among health status, personal factors, and systemic, social and environmental factors that create inequitable barriers to health and functioning.^87^ Most studies identified disability through assessments of functioning difficulty, with a majority relying on 1 or more sets of WG questions on functioning. Other studies used tools based on similar concepts and methods of measurement. Studies used WG responses in multiple ways to disaggregate data by disability status,^88^ including the use of different thresholds or cumulative scores. Disability identification methods all carry limitations. For example, WG question sets were originally designed for inclusion in surveys; however, expansion of use in other setting (eg, administrative data) has prompted numerous discussions about the limitations of these measures. The WG questions, for example, may undercount people with mild limitations or with certain limitation types such as blindness and deafness.^89^^,^^90^ Decisions on the cutoffs used to disaggregate WG responses also have implications for the groups identified. The WG recommends the use of a threshold of “a lot of difficulty”; however, this may lead to the misclassification of people with disability.^91^ This is evidenced by the significant difference in prevalence estimates in studies that used multiple cutoffs to define disability by varying levels of severity.^27^^,^^32^^,^^36^^,^^37^^,^^69^ It is crucial to select a method of disability identification that is valid, reliable, and fit for purpose, at the same time acknowledging the benefits and limitations of the selected approach. This will depend on the context in which the measure is included. Even when using the same measurement tool in multiple regions, there can be large discrepancies in the identified prevalence of disability (eg, refer to Prynn et al.^21^). This highlights complications with making intercountry comparisons, because context-specific challenges may undermine the comparability of any tool use.

Although many of the disability indicators used in the studies asked about functioning with the use of assistive devices, few explored access to these technologies or considered the implications of unmet needs. Banks et al. highlighted the importance of capturing this information, stating that “information on disability-specific indicators—such as access to…assistive devices, provision of accommodations—[is] also important to capture to provide the full picture of the needs of people with disabilities.”^13^

We found that some common disability measures were missing from the present review. For instance, the Model Disability Survey is an ICF-based disability measurement tool, which also collects details on barriers and inequities.^92^^,^^93^ Although this comprehensive tool has been used to describe characteristics of populations of people with disability, measure prevalence, and identify disability as an outcome,^94‑97^ it has not yet been used to compare health outcomes between people with and without disability in LMICs within peer-reviewed literature meeting our inclusion criteria.

Health disparities were found in almost every included study, with few studies not finding differences between people with and without disability in at least 1 of the outcomes explored. Nearly 2 decades have passed since the UNCRPD enactment and recognition of the right for people with disability to the highest attainable standard of health (Article 25).^1^ Despite recent progress reported by WHO, including the introduction of disability-specific policies and initiatives in countries (eg, the Philippines, Nepal),^3^ it is clear that people with disability in LMICs still experience disparities in morbidity, mortality, and access to health services compared with individuals without disability. Within the many health-related outcomes explored, themes of maternal and child health and health care access, as well as HIV, emerged as areas of focus, and these areas align with priority health areas of the SDGs.^7^

In the studies that explored subgroups of people with disability (eg, by impairment type or degree of disability severity), differences between groups were observed. This suggests it is beneficial, where possible, to disaggregate analyses by subgroups to more accurately identify cohorts at greater risk. Barreto et al.,^29^ for example, found that when compared with people without disability, individuals with vision impairment had a greater risk of smoking, whereas people with mental, intellectual, physical, or multiple impairments had a lower comparative risk of smoking. If these groups were only considered as an aggregate, important differential effects could be masked, leading to ineffective policy and prevention targets.^98^

Monitoring and evaluation are highlighted as a key strategic point for progress of disability inclusion and health equity by WHO.^3^ The collection and disaggregation of disability data are crucial for the development of appropriate policies and to monitor progress toward the implementation of the UNCRPD^1^ and other national targets, such as the SDGs.^7^ Most of the included studies were cross-sectional and focused on data from a single point in time. To monitor disparities and contributing factors, repeated measures over time are required so changes in outcomes and progress can be identified. Only 4 of the included studies used longitudinal data or repeated measures over time.^14^^,^^15^^,^^35^^,^^42^ Yan et al.^35^ compared depressive symptoms between older adults with and without disability in 2015 and 2018, and by analyzing data from multiple time points, they were able to demonstrate increases in depressive symptoms and disparities in the population over time. Although Casebolt et al.^30^ did aim to conduct longitudinal analysis, they were unable to do so due to the absence of a variable that could be used to link data collected in different years, which highlights how limitations with data may hinder efforts to effectively conduct monitoring and evaluation.

A wide breadth of health-related outcomes were explored in the included studies; however, there were substantial gaps in evidence. For example, few of the studies looked at vaccination for preventable disease, with only 2 studies looking at aggregate measures of vaccination coverage^47^^,^^71^ and 1 considering the influenza vaccine.^48^ Despite the proliferation of research following the outbreak of COVID-19,^99^ COVID-19 outcomes including access to vaccination—which has been highlighted in some HICs^100‑102^—was not covered in the included studies. Similarly, although there has been increasing focus on improving equity in health screening (eg, cervical cancer^103^^,^^104^), this is yet to translate to comparable evidence in LMICs. Death was explored in 2 of the studies, with findings that risk of all-cause mortality was 2 to 3 times higher for people with disability compared with those without.^14^^,^^15^ No study, however, focused on avoidable death as a result of treatable or preventable causes, which may be a more informative outcome when considering implications for policy and action.^105^

Seven studies found positive results, and these were primarily related to improved access to health services for people with disability. Although not consistent across studies, these results may indicate that program and policy implementation in recent years has improved some aspects of health and health care for people with disability. For instance, when exploring HIV-related outcomes Abimanyi-Ochom et al.^69^ found similar levels of knowledge between adolescents and adults with and without disability. Those authors suggested this may reflect concerted efforts to improve outcomes for people with disability in this area.

It can be challenging to differentiate between inequities and inequalities, because the data required to demonstrate the pervasive and unjust drivers of disparity often are lacking.^3^ For this reason, many studies comparing health outcomes between people with and without disability were only able to establish the existence of inequality but could not definitively identify inequity. In some cases, however, disparities in health outcomes can be clearly identified as inequitable—for instance, when the outcome is entirely preventable. An example of this is cervical cancer, which is entirely preventable and treatable; therefore disparities in cervical cancer outcomes can be considered inequitable. For most health outcomes, however, it was not possible to determine whether disparities between people with and without disabilities were preventable (inequities) and, therefore, are considered inequalities. Many of the studies included in this review identified health-related inequality, but future research should not only explore differences in health outcomes but also aim to identify the avoidable differences in health driven by underlying inequitable causes. This would help to identify and prioritize actionable targets to improve health equity for people with disability. Building capacity for data linkage may offer future opportunities to improve monitoring and evaluation and enable analysis of the drivers of inequity.

Improved disability data collection and program implementation are needed to address health disparities for people with disability. Although several articles discussed how the research could inform future policy and programs to reduce disparities for people with disability, only 1 study described actions taken to promote such change. Marella et al.^43^ shared findings with both government and nongovernment stakeholders to advocate for disability inclusion. Their study engaged people with disability, OPDs, government representatives and policy makers throughout the project to drive meaningful engagement and change.

Active involvement of people with disability is crucial to ensure that research is inclusive and responsive to the needs and perspectives of the community. Principles of participation have long been highlighted by the sentiment of “Nothing About Us Without Us.” This position was reiterated by Wilbur et al.,^22^ who discussed the need for policy development and implementation to be led by OPDs. We found evidence of inclusive research practice, particularly in studies conducting primary research, through the engagement of people with disability in consultation on study design methodology, the recruitment of data collectors with lived experience of disability, and development of accessible materials both for data collectors and for study participants.

### Limitations

In the protocol for this scoping review, we specified that we would include both peer-reviewed and gray literature on the topic; however, only peer-reviewed literature was included in this review. This may have resulted in the exclusion of potentially informative findings, particularly in regions where reports from governments and nongovernment organizations may be produced more frequently than peer-reviewed literature. Though some scoping reviews elect to include quality assessments, it is not a standard step in the scoping review process,^8^ because the aims of scoping reviews are often to map the breadth of available literature on a topic, and not to assess methodological quality or synthesize results in ways that may require greater understanding of the quality of included evidence. We did not conduct quality assessment of the included articles, and this may have led to the inclusion of low-quality studies in the review.

## Conclusions

There is a growing interest in using population-based data to understand the extent of health-related disparities for people with disability in LMICs. We found numerous methodological approaches; however, there was a noticeable lack of administrative data used for monitoring purposes. This may reflect barriers in resource-limited settings to implement systems that can capitalize on the potential of these data sources. The potential for use of systems that collect data continuously over time presents an opportunity to monitor longitudinal progress toward national goals of health equity for people with disability. Resources such as the WHO Disability-Inclusive Health Services Toolkit^73^ could aid in the development of HIS architecture in LMICs to support such progress. Despite the range of evidence on widespread health disparities across numerous outcomes, it is unclear how these findings have been used to enact real change. Future research should focus on disability-inclusive practice with outcomes designed to inform policy and to progress national agendas of health equity for people with disability.

## Supplementary Material

Web_Material_mxag009
