# Supplementary material for: Monitoring and evaluation of health disparities for people with disability in low- and middle-income countries: a scoping review
Source: Epidemiol Rev. 2026 Mar 24;48(1):mxag009. doi: 10.1093/epirev/mxag009 (PMC13175618; doi:10.1093/epirev/mxag009)
Supplement: Web_Material_mxag009 [file web_material_mxag009.zip › Supplementary_Tables_S1-S3.docx]

**Supplementary file: Manuscript title: Monitoring and evaluation of health disparities for people with disability in low- and middle-income countries: a scoping review**

**Authors:** Sarah Walmsley, Marie Huska, Zoe Aitken, Manjula Marella, Anne Kavanagh, Kaloyan Kamenov, Darryl Barrett, Alexandra Devine

Table of Contents

[Table S1. Search strategy for MEDLINE (Ovid) 1](#_Toc223556486)

[Table S2. Study characteristics, health-related outcomes and details of relevant findings organised by study region 3](#_Toc223556487)

[Table S3. Prevalence of disability found in included studies organised by study region 18](#_Toc223556488)

# **Table S1. Search strategy for MEDLINE (Ovid)**

| 1 | (afghan* OR ((west* OR east* OR central OR sout* OR north*) adj2 africa*) OR albania* OR algeria* OR angola* OR argentin* OR armenia* OR azerbaijan* OR bangladesh* OR belarus* OR belorus* OR byelarus* OR byelorus* OR belize* OR benin* OR dahomey OR bhutan* OR bolivia* OR bosnia* OR herzegovin* OR botswan* OR batswan* OR bechuanaland* OR brazil* OR brasil* OR bulgaria* OR burkina* OR burundi* OR cabo verde* OR cape verde* OR cambodia* OR cameroon* OR cameroun* OR chad* OR china* OR colombia* OR comoro* OR comore* OR congo* OR costa rica* OR cote d'ivoir* OR cote d' ivoir* OR cote divoir* OR cote d ivoir* OR ivory coast* OR cuba OR djibouti* OR dominica* OR ecuador* OR egypt* OR el salvador* OR guinea* OR equatoguinea* OR eritrea* OR eswatini* OR swaziland* OR ethiopia* OR fiji* OR gabon* OR gambia* OR (georgia NOT (atlanta OR california OR florida)) OR ghana* OR grenada* OR guatemala* OR haiti* OR hondura* OR india* OR indonesia* OR iran* OR iraq* OR jamaica* OR jordan* OR kazakh* OR kenya* OR karabati* OR north korea* OR DPRK OR kosovo* OR kosova* OR kyrgyz* OR kirgiz* OR kirghiz* OR laos OR lao OR lebanon* OR lesotho* OR liberia* OR libya* OR macedonia* OR madagasca* OR malawi* OR malaysia* OR maldives* OR indian ocean OR mali OR micronesia* OR kiribati* OR marshall island* OR tuvalu* OR mauritania* OR mauritius* OR mexico* OR moldova* OR moldovia* OR mongol* OR montenegr* OR morocco* OR mozambique* OR myanmar* OR namibia* OR nepal* OR nicaragua* OR niger* OR pakistan* OR palestin* OR gaza* OR west bank* OR palau OR paraguay* OR peru OR philippine* OR philipine* OR phillipine* OR phillippine* OR russia* OR rwanda* OR ruanda* OR samoa* OR pacific island* OR polynesia* OR sao tome* OR senegal* OR serbia* OR sierra leone* OR melanesia* OR solomon island* OR somali* OR sri lanka* OR saint lucia* OR st lucia* OR saint vincent* OR st vincent* OR grenadine* OR sudan* OR surinam* OR syria* OR tajik* OR tadjik* OR tadzhik* OR tanzania* OR thai* OR timor leste* OR east timor* OR togo OR tonga* OR tunisia* OR turkiy* OR turkey* OR turkmen* OR uganda* OR ukrain* OR uzbek* OR vanuatu* OR vietnam* OR viet nam* OR yemen* OR zambia* OR zimbabwe* OR arab* countr* OR middle east* OR global south OR sahara* OR subsahara* OR magreb* OR mati),ab,hw,kf |
| --- | --- |
| 2 | ((intellectual OR learning OR developmental OR physical OR sensory OR mental OR psychological OR hearing OR vision OR speech OR cognitive) AND (disabilit* OR impair*)).mp |
| 3 | exp Disabled Persons/ |
| 4 | ((disable* OR disabilit*) adj5 (person* OR people OR child* OR adult?)).mp |
| 5 | Health inequities/ |
| 6 | Health status disparities/ |
| 7 | Healthcare disparities/ |
| 8 | exp Health Behavior/ |
| 9 | Health Knowledge, Attitudes, Practice/ |
| 10 | (((health OR disability OR disability-based) AND (barrier* OR disparit* OR equal* OR exclusi* OR imped* OR inaccess* OR access* OR inequal* OR inequit* OR inhibit* OR unequit* OR utilis* OR utiliz* OR obstacle* OR gap)) OR morbidit* OR mortality).mp |
| 11 | routinely collected health data/ |
| 12 | health information systems/ |
| 13 | hospital information systems/ |
| 14 | electronic health records/ |
| 15 | health survey or health care survey/ |
| 16 | (((administrat* OR health OR population OR population-based OR management OR community* OR community-based OR hospital* OR institution OR organisation OR patient OR medical) adj (data OR record OR information)) OR survey* OR census).mp |
| 17 | 2 or 3 or 4 |
| 18 | 5 or 6 or 7 or 8 or 9 or 10 |
| 19 | 11 or 12 or 13 or 14 or 15 or 16 |
| 20 | 1 and 17 and 18 and 19 |
| 21 | Limit 20 to yr=”2008-Current” |

# **Table S2. Study characteristics, health-related outcomes and details of relevant findings organised by study region**

| **First Author, Year** | **Country/ies** | **Study cohort** | **Study design** | **Disability indicator details** | **Disaggregation by disability** | **Health-related outcome type** | **Health-related outcomes measured** | **Main findings** |
| --- | --- | --- | --- | --- | --- | --- | --- | --- |
| **East Asia and the Pacific** | | | | | | | | |
| He et al. 2019 | China | ≥45 years | Cohort study | Katz Index of Independence in ADL | Participants were asked if they had difficulty taking a bath, eating, getting in and out of bed, dressing, using the toilet, and defecating. Disability was categorised into two levels: difficulty in one ADL, and difficulty in two or more ADL. Spouses of people with disability were included and were also categorised into the two levels based on their partner’s level of disability. | Morbidity | Depression | A higher risk for depression was found for people with ADL scores of two or more, compared to people without disability. People with ADL scores of one also had higher risk of disability, however this association did not remain after adjustment. A similar pattern was found for spouses of people with disability, with those who had spouses with ADL scores of two or more at greater risk of depression. |
| Shi et al. 2024 | China | ≥60 years | Cross-sectional survey | Multiple questions^a^ | Participants were asked multiple questions regarding independent living, mobility, speaking, hearing and memory over the past 6 months. Disability was identified if individuals reported not being able to live independently, any mobility issues, any problem speaking, any difficulty hearing or significant memory decline. | Access; Knowledge/  attitudes/  practice | Utilisation of community healthcare centre, utilisation of home health services from community healthcare centre, satisfaction with community healthcare centre | Older adults with mobility and cognitive disability were more likely to receive home health services than older adults without disability. No statistically significant associations were found for overall disability status and community healthcare centre use or satisfaction. |
| Yan et al. 2023 | China | ≥60 years | Cohort study | Multiple questions (physical function) ^a^; ADL scale^a^ | Physical function disability was defined as a participant reporting difficulty with any of the 9 physical function items (running or jogging 1km, wandering 1km, walking 100 meters, sitting in a chair for a long time and then standing up, ascending several floors continuously, bending over, bending knees or squat, stretch arms up along your shoulders, walk 100 meters to run or jog 1 km, pick up a tiny coin from the table). ADL disability was defined as having difficulty with any of the 12 ADL items (dressing, bathing, eating, getting into or out of bed, using the bathroom, controlling urination and defecation, doing household chores, cooking, shopping, making phone calls, taking medication, managing money). | Morbidity | Depression | Physical function and ADL were significantly associated with presence of depressive symptoms. These associations varied by rurality with both physical function and ADL disability being associated with depressive symptoms for rural older adults, while only ADL disability being associated with depressive symptoms for urban older adults. |
| Marella et al. 2016 | Philippines | ≥18 years | Cross-sectional survey | Rapid Assessment of Disability survey | Participants were asked about difficulty in eight functional domains: vision, hearing, communication, mobility, gross and fine motor, cognitive, appearance, and psychological distress. Disability was defined as having difficulty 'most' or 'all of the time' even with the use of assistive devices in at least one item from the first seven domains, or at least two items from the psychological distress domain. | Access; Other: Well-being | Well-being, access to the community | Disability was significantly associated with low well-being scores. People with disability were also more likely to experience reduced access to health services, work, rehabilitation, education, government social welfare and disaster management than people without disability. |
| Wilbur et al. 2021 | Vanuatu | ≥5 years | Mixed methods | WG Short Set | Participants were asked about difficulty in six functional domains: seeing, hearing, walking, cognition, self-care, and communication. Disability was defined as a response of 'a lot of difficulty' or 'cannot do at all' in at least one domain. | Morbidity | Incontinence | People with disability were three times more likely to experience incontinence than people without disability. People with disability were found to be less able to wash and participant in social activities than people without disability. Limited access to assistive technologies and lifting devices for caregivers were also found. |
| **Latin America and the Caribbean** | | | | | | | | |
| Grushka et al. 2020 | Argentina | ≤99 years | Cohort study using administrative data | Administrative data item (receipt of disability pension) | People with disability were identified through disability pension administrative records. Criteria for the receipt of disability pension included impact on capacity to work and was only available to those considered to have severe disability. | Mortality | Mortality | The relative risk of mortality was twice as high for people with disability compared to the country standardised mortality rate. There was a more than 13-year life expectancy gap for both females and males with disability at age 40, however this gap was reduced by age 65. Risk of mortality was inversely related to duration of disability support pension, indicating that risk of mortality was highest during the initial years of disability support pension receipt. |
| Barreto et al. 2023 | Brazil | ≥15 years | Cross-sectional survey | Multiple questions^a^ | An answer of to any of the impairment related questions related to hearing, visual, physical, cognitive, or intellectual impairment. | Morbidity | Cardiovascular disease, hypertension, diabetes mellitus, high cholesterol, alcohol abuse, smoking | People with one or more impairment had greater odds of chronic conditions, particularly cardiovascular disease and diabetes mellitus, as well as health risk behaviours. Visual impairment was associated with higher odds of smoking. Cognitive impairment, intellectual impairment, physical impairment and multiple impairments were associated with lower odds of smoking and alcohol abuse. |
| Macarevich Condessa et al. 2021 | Brazil | ≥18 years | Cross-sectional survey | Multiple questions^a^ | Disability was defined as answering yes to any question when asked “Do you have an intellectual disability” “Are you physically disabled” “Is you hearing impaired?” and “Do you have any visual impairment?” | Access | Use of dental healthcare services | No statistically significant associations were found between dental healthcare service use and disability. In unadjusted models people with disability were found to visit the dentist more regularly, however after adjusting for health behaviour and oral health conditions the association was no longer significant. |
| Silva et al. 2017 | Brazil | ≥60 years | Cross-sectional survey | Multiple questions^a^ | Participants were asked about the degree of difficulty performing six basic activities (feeding, bathing, using the toilet, dressing, walking at home from one room to another on the same floor, and lie down or get up from bed) and four instrumental ADL (shopping, managing own finances, taking medications, and leaving home using transportation), with disability defined as those who reported some degree of difficulty in at least one of the activities measured. | Access | Use of health services, quality of medical care received | Functional limitations were significantly associated with number of doctor visits and hospitalisations in the last 12 months for both public and private systems. People with functional limitations using the public system reported worse freedom in choosing a doctor and longer waiting times for appointments than public system users without functional limitations. |
| Lopez-Gil et al. 2021 | Colombia | 13-17 years | Cross-sectional survey | Multiple questions^a^ | Vision impairment was defined as reading or writing problems such as image distortion, tiredness, red eyes, skipped lines or reversed letters and numbers. Hearing impairment was defined as any ear or hearing problem in the last 30 days as well as having ever been told by a health progressional that they have a hearing or ear problem. | Knowledge/  attitudes/  practice | Physical activity, screen time | Males with hearing or vision impairment, and females with vision impairment had lower odds of meeting the physical activity recommendations. Females with vision impairment and hearing impairment, and males with vision impairment had lower odds of meeting screen time recommendations |
| Kuper et al. 2018 | Guatemala | ≥2 years | Nested case-control study | WG Extended Set; WG/UNICEF CFM | Participants aged 10 years or older, and caregivers of children aged under 10 years as a proxy, were asked about difficulty in functional domains. Disability was defined for adults as a response of ‘a lot of difficulty’ or ‘cannot do’ in at least one domain of seeing, hearing, walking, self-care, communication, cognition, upper body dexterity/strength, or ‘a lot’ of anxiety/depression daily. For children aged 2-4 years disability was defined as a response of ‘a lot of difficulty’ or ‘cannot do’ in at least one domain of seeing, hearing, walking, fine motor dexterity, understanding, being understood, learning, playing and/or controlling behaviour. For children aged 5-17 years disability was defined as a response of ‘a lot of difficulty’ or cannot do’ in at least one domain of seeing, hearing, walking, self-care, understanding, being understood, learning, remembering, concentrating, accepting change, controlling behaviour, anxiety and/or depression. | Access; Morbidity | Access to healthcare and rehabilitation services, conditions and coverage of treatment for conditions, healthcare seeking and experience | People with disability had higher odds or reporting a serious health problem or having received a health condition diagnosis from a doctor than people without disability. People with disability were two times more likely than people without disability to receive treatment for a diagnosed health condition. People with disability were more likely to report being disrespected during their last healthcare visit, and finding it difficult to understand information given more than people without disability. |
| Casebolt 2024 | Haiti | Women 15-49 years | Cross-sectional survey | WG Short Set | Weighted scores were given to answers of difficulty with the six core domains of seeing, hearing, walking, remembering, communicating, and completing self-care activities were weighted (no difficulty = 0; some difficulty = 1; a lot of difficulty = 6; cannot do at all = 36). Disability was categorised as no disability (0), mild disability (1-4), moderate disability (5-23) and severe disability (24-216). | Morbidity | HIV morbidity | Associations between HIV and disability varied by disability severity. Those with mild disability had higher odds of HIV infection than those without disability, however those with moderate and severe disability did not have higher odds of HIV infection than those without disability. |
| Danquah et al. 2015 | Haiti | ≥5 years | Nested case-control study | WG Short Set | Participants were asked about difficulty in six functional domains: seeing, hearing, walking, cognition, self-care, and communication. Disability was defined as a response of 'some difficulty' in at least two domains or 'a lot of difficulty' 'cannot do at all' in at least one domain. | Access; Knowledge/  attitudes/  practice | Number of visits to health centre in the last year, difficulty accessing facility, barriers accessing health services/medical care | Adults with disability were more likely to visit health services than those without disability. Difficulty in accessing health facilities was found to be higher for boys with disability compared to boys without disability, this same association was not found for girls. |
| de Castro et al. 2017 | Mexico | 2-17 years | Cross-sectional survey | WG/UNICEF CFM | Mothers were asked if their child aged 2-4 years had difficulty in 9 functional areas: seeing, hearing, walking, understanding, being understood, learning things, learning names, playing, and controlling behaviour. Disability was defined as ‘a lot of difficulty’ or ‘cannot do at all’ in at least one of the first 9 functional areas, or ‘much more’ for controlling behaviour. Children and adolescents aged 5-17 years were asked if they had difficulty in 14 functional areas: seeing, hearing, walking, feeding or dressing, being understood inside the household, being understood outside the household, learning, remembering, focusing, accepting change, and making friends, anxiety, depression, controlling behaviour. Disability was defined as ‘a lot of difficulty’ or ‘cannot do at all’ in at least one of the first 11 domains, ‘daily’ anxiety or depression, or ‘much more’ for controlling behaviour. | Access; Morbidity | Sustainable Development Goal outcomes: Stunting, underweight, full vaccination coverage, early childhood development, use of improved drinking water sources, use of improved sanitation, place for handwashing, child labour, violent discipline, birth registration | Children with severe functional disability were more likely to be underweight, and less likely to have adequate early child development, compared to children without severe functional disability as well as national levels. No significant differences were found between children with and without severe functional difficulties in terms of stunting, full vaccination coverage, and violent discipline. |
| Flores-Flores et al. 2018 | Peru | 65-80 years | Cross-sectional survey | Katz Index of Independence in ADL | Disability was defined as permanent difficulty lasting 3 months or more reported at the level of “much difficulty” performing for any of the six activities: dressing, feeding, transferring in and out of bed, walking, bathing, and using the toilet. | Access | Having health insurance, having received specific preventative services (vision assessment, influenza vaccination, blood pressure assessment, diabetes screening, cholesterol assessment) | People with ADL disability were 63% less likely to have extensive health insurance compared to people without disability. This association was modified by sex, with men with disability being more likely than men without disability to have insurance whereas women with disability were significantly less likely to have health insurance than women without disability. Those with disability were more likely to have received a blood pressure assessment in the last two years compared to people without disability. No other significant associations were found for preventative services. |
| Hernandez-Vasquez et al. 2023 | Peru | Migrants and refugees from Venezuela, ≥18 years | Cross-sectional survey | WG (set not specified) | Participants were asked to answer yes or no to questions about whether they experienced difficulty in six functional domains: walking, seeing, communicating, hearing, cognition, emotions. Disability was defined as a response of yes to at least one domain. | Other: Unmet food needs | Unmet food needs | The study found that people with disability were more likely to have an unmet need for access to food at home compared to people without disability. |
| Rohrer et al. 2010 | Peru | ≥18 years | Cross-sectional survey | Single question | Participants self-reported disability in response to the question “are you limited in any way in any activities because of physical, mental or emotional problems?” | Other: Self-rated health | Self-rated health | Self-reported disability was found to be negatively associated with good self-rated health, after adjusting for age and gender. Joint pain was also found to be inversely related to good self-rated health. |
| **Middle East and North Africa** | | | | | | | | |
| Trani et al. 2018 | Morocco and Tunisia | Whole population | Case-control study | Disability Screening Questionnaire (DSQ-34) | Severe disability was defined as an answer of ‘yes, often’ or ‘constantly, always’ to at least one of the 34 items in the questionnaire (35 items for children). | Access | Access to healthcare, out-of-pocket expenditures, health related quality of life, multidimensional poverty, employment/type, access to school and literacy | In both countries people with disability were found to have lower health related quality of life, and higher risk of multidimensional poverty. In Tunisia no effect found on access to health care and expenses, however in Morocco disability was associated with reduced access to healthcare facilities and higher out of pocket expenditure. |
| **South Asia** | | | | | | | | |
| Casebolt et al. 2022 | India | Women 15-49 years | Cross-sectional survey | Single question | Women were asked to report if they had any form of disability, with all disability types combined into a single dichotomous disability variable. Disability types were measured and categorised into types based on the highest functional impact, however resulting prevalence was too low for analyses based on type. | Knowledge/  attitudes/  practice | Use of modern contraceptive methods, female sterilisation | Women with disability were less likely to report use of modern contraceptives than women without disability. Following adjustment, women with disability did not have higher odds of sterilisation than women without disability. |
| Casebolt et al. 2023 | India | Women 15-49 years | Cross-sectional survey | Single question | Women were asked to report if they had any form of disability, with all disability types combined into a single dichotomous disability variable. Disability types were measured and categorised into types based on the highest functional impact, however resulting prevalence was too low for analyses based on type. | Access | Antenatal care, skilled delivery, postnatal care | Women with disability were less likely to report attending the minimum antenatal care visits than women without disability. No association was found between disability and skilled delivery or postnatal care. |
| Grills et al. 2017 | India | ≥18 years | Case-control study | Rapid Assessment of Disability survey | Defined as ‘at risk of disability’ and included participants who reported difficulties most or all of the time in at least one domain of vision, hearing, communication, mobility, gross and fine motor skills, cognition, and appearance, or at least two of the six items on psychological distress. | Access; Other: Well-being | Access to the community (health, education, work, social, legal, religious, rehabilitation, other services), well-being (good health, making friends, being safe in daily life, taking care of self) | People with disability were found to have significantly less access to the community than people without disability. Identified barriers included lack of information, transport and physical access barriers. People with disability had more than five times higher odds of reporting unmet health service needs than people without disability. The odds of unmet rehabilitation needs were also found to be two times higher for people with compared to people without disability. |
| Gudlavalleti et al. 2014 | India | ≥18 years | Case-control study | Assessment by key-informant with confirmation by trained investigator | Initial listing of disability was identified by key-informants and therefore was defined as impairments which were identified by visible assessment and taking a short medical history, supported by information from disability certificates and disability support pension records. Disability status was then confirmed with follow-up by medical physicians and therapists. | Access; Morbidity | Health care access and issues (hospital visit in last year, hospitalisation experience, current medication, chronic disease presence and duration, health service access barriers, quality of care) | In unadjusted analysis people with disabilities needed to visit hospitals more often in the preceding year, however this became statistically insignificant after adjustment. Past hospitalisation, risk of diabetes, and risk of depression were all found to be higher for people with compared to people without disability. People with disability also reported more barriers accessing health services including accessibility of services, costs of services, and transportation. |
| Mathias et al. 2018 | India | ≥18 years | Nested case-control study | Rapid Assessment of Disability (psychosocial disability section only, based on a modified version of the Kessler-6) | Disability was defined as a response that the participant had difficulties 'most of the time' or 'all of the time' in at least two domains of the Kessler-6. | Access | Access to the community (employment, health services, community consultations, disabled persons organisations, social activities, sanitation, safe drinking water, religion) | Unmet service access needs were significantly higher for people with psychosocial disability. People with psychosocial disability were found to encounter greater barriers in every domain compared to people without disability; especially in the domains of employment, health service access and community consultation. |
| Muhammad et al. 2022 | India | ≥60 years | Cross-sectional survey | Single question (vision impairment); ADL scale^a^; IADL scale^a^ | Vision impairment: if the participant reported any difficulty with vision. ADL: Potential scores ranged from 0 to 6, a score of 5 or less was defined as disability. IADL: Potential scores ranged from 0 to 8, a score of 5 or less was defined as disability. | Morbidity | Vision impairment, cognitive impairment | Those with vision impairment were found to be 11% more likely to have cognitive impairment than those without vision impairment. Low independence for ADL and low independence for IADL were found to be risk factors for cognitive impairment. |
| Murthy et al. 2014 | India | Women 15-45 years | Case-control study | Assessment by key-informant with confirmation by trained investigator | Initial listing of disability was identified by key-informants and therefore was defined as impairments which were identified by visible assessment and taking a short medical history, supported by information from disability certificates and disability support pension records. Disability status was then confirmed with follow-up by medical physicians and therapists. | Access; Morbidity | Diabetes, depression, mental health issues, maternal health issues including pregnancy history, delivery, complications, receipt of healthcare and advice | Women with disability were more likely to have diabetes and depression, and were less likely to have experienced pregnancy, than women without disability. A lower proportion of women with disability reported successful pregnancy in the last 2 years compared to women without disability. Similar levels of antenatal care utilisation were found for women with and without disability. |
| Devkota et al. 2017 | Nepal | Women 15-49 years | Case-control study | WG Short Set | Described as meeting WG criteria for disability. | Access; Knowledge/  attitudes/  practice | Outcomes related to health facilities, healthcare delivery, inter-personal aspects of healthcare providers, the accessibility of services | The study found a difference in the perceived quality of care in health facilities between women with and women without disability, and these differences were not linked to caste differences. Women with disability reported higher mean scores of perceived quality of care in terms of health facility cleanliness and facilities, openness and friendliness and compassion and kindness of staff. Women without disability reported higher mean scores of perceived availability of cash incentives to access services. |
| Devkota et al. 2021 | Nepal | Women 15-49 years | Case-control study | WG (set not specified) | Described as meeting WG criteria for disability. | Access | Maternal healthcare service utilisation (antenatal care, health facility delivery, postnatal care during last pregnancy) | No significant associations were found between maternal healthcare service utilisation and disability. Women with a disability had lower odds of delivering their baby in a healthcare facility compared to women without a disability in unadjusted analysis, however after adjusting for age this association was no longer found. |
| Hameed et al. 2023 | Pakistan | Women 15-49 years | Cross-sectional survey | WG Short Set | Women were asked about difficulty in six functional domains: seeing, hearing, walking, cognition, self-care, and communication. Disability was defined as ‘a lot of difficulty’ or ‘cannot do at all’ in at least one domain. At risk of disability was defined as ‘some difficulty’ in one or more domains. | Access | Utilisation of essential ANC for most recent birth (including ANC coverage, treatment, examination) | There was no difference found in ANC coverage between women with and women without disability. Women with disability were more likely to receive some components of essential ANC including receiving advice on exclusively breastfeeding and receiving urine tests. Women at risk of disability were found to have higher odds of receiving advice around balanced diet than women not at risk of disability. |
| Mahmood et al. 2022 | Pakistan | Women 15-49 years | Cross-sectional survey | WG Short Set | Participants were asked about difficulty in six functional domains: seeing, hearing, walking, cognition, self-care, and communication. Disability was defined as a response of 'some difficulty' 'a lot of difficulty' or 'cannot do at all' in at least one domain. Severe disability was defined as a response of 'a lot of difficulty' or 'cannot function at all' in at least one domain. | Access | Modern contraceptive use, skilled antenatal care, skilled birth attendance, skilled postnatal care for last live birth | No statistically significant differences were found between women with and without disability in adjusted model. In unadjusted analysis women with disability were less likely to use antenatal care, skilled birth attendance, and skilled postnatal care than women without disability, however these associations did not remain following adjustment. |
| Banks et al. 2020 | The Maldives | ≥2 years | Nested case-control study | WG Extended Set; WG/UNICEF CFM; Registration with National Social Protection; Receipt of Disability Allowance | Participants, or proxies for children under 10, were asked whether they experienced difficulty in a range of functional domains. Disability was defined as a response of ‘a lot of difficulty’ or ‘cannot do at all’ in at least one domain. For domains anxiety and depression children aged 5 years or older who experienced symptoms at frequency of ‘daily’ and adults aged 18 years or older who experienced the same frequency of symptoms at an intensity of ‘a lot’ were also defined as having disability (for the case-control part of the study a report of functional impact on work, schooling, or social relations was also required to identify cases). Cases were also identified through registration with Maldivian Government National Social Protection, for the receipt of Disability Allowance for example. | Access; Morbidity | Sustainable Development Goals based outcomes (household medical expenditure in the last month, serious health problem in the last 12 months) | People with disability were two and a half times more likely to experience a serious health problem in the last 12 months compared to people without disability. Among people with disability the risk of serious health problems was highest among people with mental health difficulties. No difference was found for catastrophic health expenditure among households with people with disability compared to households without people with disability. |
| Banks et al. 2022 | The Maldives | ≥2 years | Nested case-control study | WG Extended Set; WG/UNICEF CFM; Receipt of Disability Allowance; Self-reported health condition | Participants, or proxies for children under 10, were asked whether they experienced difficulty in a range of functional domains. Disability was defined as a response of ‘a lot of difficulty’ or ‘cannot do at all’ in at least one domain. For domains anxiety and depression children aged 5 years or older who experienced symptoms at frequency of ‘daily’ and adults aged 18 years or older who experienced the same frequency of symptoms at an intensity of ‘a lot’ were also defined as having disability (for the case-control part of the study a report of functional impact on work, schooling, or social relations was also required to identify cases). Cases were also identified if they received the Disability Allowance or had a medical condition which made them eligible for receipt. | Access; Morbidity | Indicators of general health and healthcare access (diagnosis and treatment of chronic conditions, self-rated health, serious health event in last 12 months, healthcare access, challenges, cost, insurance) | People with disability were found to have poorer levels of health and self-rated health, higher likelihood of chronic conditions, and higher likelihood of serious health events in the previous 12 months compared to people without disability. People with disability were also more likely to report difficulty with healthcare access than people without disability. Access to disability-related healthcare varied by disability type. |
| **Sub-Saharan Africa** | | | | | | | | |
| Onadja et al. 2013 | Burkina Faso | ≥15 years | Cross-sectional survey | WG Short Set | Participants were asked about difficulty in six functional domains: seeing, hearing, walking, cognition, self-care, and communication. Responses were coded as 0 no difficulty, and 1 any difficulty. A summative score of all six functional limitations was calculated, and categorised as no limitation (0), one limitation (1), or two or more limitations (2+). | Other: Self-rated health | Self-rated health | Functional limitations were strongly associated with poor self-rated health. The association between functional limitations and poor self-rated health increased with age and was stronger for those who were less educated. |
| DeBeaudrap et al. 2019 | Cameroon | 15-49 years | Case-control study | WG Extended Set | Participants were asked about difficulty in six basic functional domains (seeing, hearing, walking, cognition, self-care, and communication) as well as two domains from the Extended Set (learning and analysing). Those who reported difficulty in physical, visual and hearing domains were included. Disability was disaggregated into mild, severe and total. | Access; Knowledge/  attitudes/  practice | Use of maternal care, satisfaction, difficulties accessing sexual and reproductive health services, use of modern methods of family planning, use of HIV testing services | People with disability were more likely to report difficulty accessing sexual and reproductive health services than people without disability. Women with disability were more likely to have never accessed any sexual and reproductive health service than women without disability. Difficulties with accessing SRH were highest for those with mobility, hearing and severe limitations. Health worker attitudes were the most commonly reported reason for difficulties with access. HIV testing rates and use of modern contraception methods were lower among people with disability. |
| Rotenberg et al. 2023 | Central African Republic, Chad, Democratic Republic of Congo, Gambia, Ghana, Lesotho, Madagascar, Malawi, Sierra Leone, and Togo | 2-4 years | Cross-sectional survey | WG/UNICEF CFM | Caregivers were asked about whether the child experienced difficulty in eight functional domains: vision, hearing, mobility, communication/ comprehension, behaviour, learning, dexterity and playing. Disability was defined as a response of 'a lot of difficulty' or 'cannot do at all' in at least one of domains. | Access; Morbidity | Acute respiratory infection, diarrhoea and fever as well as caregiver care seeking for these illnesses | Children with disability had greater odds of acute respiratory infection, diarrhoea and fever than children without disability. No difference was found in the rate of care seeking for these illnesses between caregivers of children with disability and children without disability. |
| Massetti et al. 2024 | Lesotho | 13-24 years | Cross-sectional study | WG Short Set^b^ | Participants were asked about difficulty in six functional domains: vision, memory or cognition, walking or mobility, self-care (washing or dressing, independent living (doing chores or errands alone), and communication. Disability was defined as a response of 'some difficulty' 'a lot of difficulty' or 'cannot do at all' in at least one domain. | Knowledge/  attitudes/  practice; Morbidity;  Other: Sexual violence | HIV status, sexual risk behaviours, sexual violence | Females with a disability had higher odds of being HIV positive, having transactional sex and experiencing any lifetime violence, emotional violence, physical violence, witnessing inter-parental violence, and witnessing community violence than females without disability. Males with disability had higher odds of transactional sex, having multiple recent sex partners, experiencing emotional violence, and witnessing inter-parental violence than males without disability. |
| Carew et al. 2019 | Liberia | Whole population | Case-control study | Identification through OPD lists; Sensitivity analysis using WG Short Set | Participants with disability were identified through a list from an OPD. WG questions were used to define disability in sensitivity analysis, with disability defined as a response of ‘with a lot of difficulty’ or ‘cannot do at all’ in at least one functional domain: seeing, hearing, walking, concentration, self-care, and understanding | Other: Well-being | Multidimensional well-being | People with disability were found to experience lower life satisfaction, transport access, political participation and social inclusion compared to people without disability. In sensitivity analysis the authors removed the 70 controls that fit the Washington Group questions definition of disability (defined as a lot of difficulty or cannot do at all in at least one domain). There were only minor changes, mainly related to perceived relationship quality. |
| Devendra et al. 2013 | Malawi | 2-9 years | Case-control study | WHO Ten Question Screen (TQS) | Ten questions were used to assess presence of disability, which included delayed developmental milestones, difficulties with sight, hearing, learning/comprehension, movements, speech or seizures. Disability was defined as a positive response provided by the caregiver to any of the questions. | Access; Morbidity | Paediatric quality of life, HIV stage, history of tuberculosis, disability related access barriers | Children with disability scored significantly worse than children without disability in all domains of paediatric quality of life. HIV-positive children with disability were more likely to be at a higher WHO HIV-AIDs stage, to have had previous treatment for tuberculosis and to have below-average school grades compared to HIV-positive children without disability on enrolment. |
| Prynn et al. 2020 | Malawi | ≥18 years | Cohort study | WG Short Set | Participants were asked about difficulty in six functional domains: seeing, hearing, walking, cognition, self-care, and communication. Disability was defined as a response of 'some difficulty' 'a lot of difficulty' or 'cannot do at all' in at least one domain. | Mortality | All-cause and cause specific mortality | All-cause mortality was almost three times higher for people with disability compared to people without disability. Associations were also found between disability and higher risk of mortality from non-communicable diseases. Individual domains of self-rated walking, cognition, self-care and communication disability were strongly associated with increased mortality for all types. This association was not found for seeing and hearing disability. |
| Aderemi et al. 2013 | Nigeria | 12-19 years | Cross-sectional survey | Raven's Progressive Matrices (participant); Draw-A-Person Test (participant); Vineland's Social Maturity Scale (caregivers of learners with mild/moderate intellectual disabilities) | Not described. | Knowledge/  attitudes/  practice | Sexual behaviours, sources of HIV information, HIV transmission knowledge and substance use | More learners with intellectual disability compared to learners without intellectual disability reported having sexual experiences. Among sexually experienced female learners, those with intellectual disability had a higher prevalence of history of rape compared to those without intellectual disability. Learners with intellectual disability had lower HIV transmission knowledge scores, were less likely to have heard about HIV from common sources, and were more likely to report inconsistent condom use with boyfriends/girlfriends, casual partners and during last sexual activity compared to learners without intellectual disability. |
| Ekman et al. 2024 | Sierra Leone | 2-4 years | Cross-sectional survey | WG/UNICEF CFM | Primary caregivers were asked if the child experienced functional disability in domains of seeing, hearing, mobility, dexterity, communication, learning, behaviour and playing. Disability was defined as 'a lot of difficulty' or 'cannot do at all' in at least one of the domains. Severe functional disability was defined as a response of 'cannot do at all' in any of the domains or 'a lot more' for controlling behaviour. | Access; Morbidity | Diarrhoea, fever, acute respiratory infection, whether caregivers sought advice or treatment for the illness from any source, if child was given treatment during the illness | Children with functional difficulties had 30% higher odds of fever than children without functional difficulties, and this association increased to 60% for children with severe functional difficulties. Children with severe functional difficulties were also found to have higher odds of diarrhoea than children without functional difficulties. |
| Trani et al. 2011 | Sierra Leone | ≥18 years | Case-control study | ICF based 35-item screening tool | The 35-item tool screened for motor or physical disability, sensory disability, learning and developmental disability, behavioural disability, mood and affect disability and neurological disability, and was measured on a Likert-scale with possible answers of ‘no, never’, ‘yes, sometimes’, ‘yes, often’ or ‘yes, constantly/always. Mild to moderate disability was defined as between one to three answers of ‘yes, sometimes’ or one answer of ‘yes, often’. Severe and very severe were defined as more than three answers of ‘yes, sometimes’, more than one answer of ‘yes, often’ or at least one answer of ‘yes, constantly’. | Access; Knowledge/  attitudes/  practice; Other: Self-rated health | Self-rated health, access to public and private health care facilities, food intake and access to managed source of water and sanitation, use of contraception, access to maternal health services, number of children, desire for another child | People with disability were found to have less access to public health care services than people without disability. Women with disability were more likely to report access to maternal services than women without disability, with findings that socioeconomic factors were the main driver of inequity in this area. No differences were found for contraception use, desire for a child and having children. |
| Moodley et al. 2015 | South Africa | ≥18 years | Cross-sectional survey | WG Short Set | Not described. | Access; Morbidity; Other: Self-rated health | Self-rated health, tuberculosis, heart problems, asthma, stroke, diabetes, psychiatric disorders, epilepsy, emphysema, Alzheimer's, medical aid coverage, place of last health consultation | Compared to people without disability people with disability reported higher incidence of communicable diseases, especially tuberculosis, and higher incidence of non-communicable disease including diabetes, stroke, asthma, and heart problems. People with disability also had lower access to medical insurance and greater use of public health care than those without disability. |
| Mutwali et al. 2019 | South Africa | ≥18 years | Cross-sectional survey | WG Short Set | Participants were asked about difficulty in six functional domains: seeing, hearing, walking, cognition, self-care, and communication. Disability was defined as a response of 'some difficulty' 'a lot of difficulty' or 'cannot do at all' in at least one domain. | Access; Knowledge/  attitudes/  practice | Utilisation of healthcare, physical accessibility to healthcare facilities | Analyses were run at the household level (households with members who had disability, and households without a member who had disability). Households with members who had disability were more likely to have worse physical access to healthcare. For households with people with disability reasons for physical access barriers included lack of medical aid, difficulties associated with the predominant use of public healthcare facilities, walking to the health facility as the main form of transportation and long travel times. |
| Myezwa et al. 2016 | South Africa | ≥18 years | Cross-sectional survey | WHO Disability Assessment Schedule (WHODAS 2.0) | A combined weighted score was used to assess functional impairment in the areas of mobility, self-care, participation, cognition, getting along, life activities. Disability was defined as a weighted score of two or more. | Knowledge/  attitudes/  practice; Morbidity | Depression, adherence to antiretroviral therapy | Among people living with HIV on long-term antiretroviral medication those with disability were more likely to experience depressive symptoms and were less likely to adhere to antiretroviral therapy than those without disability. In subdomains of disability including mobility, life functionality, cognition, participation in social activities, self-care, but not getting along with other people, those who experienced depressive symptoms reported higher levels of functional limitation. |
| Pengpid et al. 2019 | South Africa | ≥15 years | Cross-sectional survey | Multiple questions^a^ | Participants self-reported disability in response to the question “Do you have any disability?” and then were asked to report the type of disability (physical, sight, hearing, communication or speech, and mental or psychiatric illness). | Knowledge/  attitudes/  practice; Morbidity | HIV and antiretroviral status, HIV/AIDS knowledge, HIV/AIDS stigma, sexual risk behaviour, intimate partner violence, psychological distress | No association was found between the prevalence of HIV infection and overall disability. When looking at disability types prevalence of HIV infection was found to be higher for people with visual and hearing disability, speech disability and hearing disability alone than for people without disability. Amongst those who were HIV positive antiretroviral exposures was found to be higher for people with disability than people without disability. People with disability had higher odds of psychological distress, having had casual or transactional sex, and having had two or more sexual partners in the last 12 months than people without disability. |
| Vergunst et al. 2017 | South Africa | ≥5 years | Case-control study | WG Short Set | Participants were asked about difficulty in six functional domains: seeing, hearing, walking, cognition, self-care, and communication. Disability was defined as a response of 'some difficulty' in at least two domains or 'a lot of difficulty' ‘unable to do’ in at least one domain. For disability severity a scale was calculated by summing scores across the 6 domains with scores ranging from 6 to 24. | Access | Health care and medical care access and barriers | People with disability had a higher rate of unmet health needs compared to people without disability, and these inequities were found to be greater for those with more severe disability. |
| Vergunst et al. 2019 | South Africa | ≥5 years | Case-control study | WG Short Set | Participants were asked about difficulty in six functional domains: seeing, hearing, walking, cognition, self-care, and communication. Disability was defined as a response of 'some difficulty' in at least two domains or 'a lot of difficulty' 'cannot do at all' in at least one domain. For disability severity a scale was calculated by summing scores across the 6 domains, with scores ranging from 6 to 18. | Knowledge/  attitudes/  practice; Morbidity | General physical health, general mental health, psychological morbidity, attitudes towards health care | People with disability reported poorer health outcomes than people without disability. Increased disability severity was associated with increased mental health problems. A higher percentage of people with disability did not receive health care when required, compared to people without disability, with the highest reason for this being associated cost. People with disability also had less trust in treatment, and were less likely to report that they perceived the health personnel at their local clinic or hospital as competent. |
| Eide et al. 2015 | Sudan, Namibia, Malawi, and South Africa | Whole population | Case-control study | WG Short Set | Responses to six functional categories (seeing, hearing, walking or climbing steps, remembering or concentrating, self-care and communication) were rated on a scale from no difficulty to unable to do. An activity limitation scale was calculated by adding the scores, with potential scores ranging from 1 to 4. A score of 4 indicated that the participant could not perform any of the activities. | Access | Health care service access, health care barriers | Activity limitation levels were associated with increased probability of not receiving necessary health care. Compared to those with no activity limitations, those with any level of activity limitations were more likely to report problems with availability of health care in the past 12 months. Reported health care access barriers included lack of transport, lack of service availability, inadequate equipment or drugs, and visit costs. |
| Chipanta et al. 2023 | Tanzania | ≥15 years | Cross-sectional survey | WG Short Set | Participants were asked about difficulty in six functional domains: seeing, hearing, walking, cognition, self-care, and communication. Disability was defined as a response of 'some difficulty' 'a lot of difficulty' or 'cannot do at all' in at least one domain. | Access; Knowledge/  attitudes/  practice; Morbidity | HIV prevalence, awareness of HIV-positive status, access to antiretroviral therapy, HIV viral load suppression | HIV prevalence was found to be higher for people with disability compared to those without. Among people living with HIV, people with disability were found to have a higher odds of knowing their HIV-positive status than people without disability. Among men living with HIV those with a disability were less likely to have supressed HIV viral loads than men without a disability. |
| Abimanyi-Ochom et al. 2017 | Uganda | Women 15-49 years, men 15-54 years | Cross-sectional survey | WG Short Set | Participants were asked if they had difficulty due to a health problem doing activities areas in six functional areas: seeing, hearing, walking or climbing steps, remembering or concentrating, self-care. Single disability was defined as at least “some” difficulty in one functional area. Multiple disability was defined as at least “some” difficulty in two or more functional areas. Severe disability was defined as “a lot of difficulty” or “cannot do at all” in at least one functional area. Hearing disability was defined as hearing difficulty even with the use of hearing aids. | Knowledge/  attitudes/  practice | HIV/AIDS knowledge, transmission and prevention methods, sexual behaviour | Women with multiple disabilities were found to be less likely to return for receipt of most recent HIV test results. People with disability reported first sexual contact at a younger age and were found to have significantly higher rates of sexually transmitted disease in the last 12 months than people without disability. People with disability reported similar levels of knowledge on HIV/AIDS transmission, although some gaps existed. |
| Kwagala et al. 2021 | Uganda | Women 15-49 years | Cross-sectional survey | WG Short Set | Participants were asked about difficulty in six functional domains: sight, hearing, speech, memory, walking, and personal care. Disability was defined as a response of 'a lot of difficulty' or 'cannot do at all' in at least one domain. | Other: Early childbearing | Early childbearing (conception or delivery before 18 years) | Early pregnancy was found to be higher among women with disability compared to women without disability. The association varied by other factors including marital status, education, occupation, and religion. Higher levels of education reduced the odds of early pregnancy for women with disability. |
| Zandam et al. 2021 | Uganda | Women 15-49 years | Cross-sectional survey | WG Short Set | Women were asked about difficulty in six functional domains: seeing, hearing, walking, remembering, communicating and, washing or taking care of self. Disability was defined as a response of 'some difficulty' 'a lot of difficulty' or 'cannot do at all' in at least one domain. | Access; Knowledge/  attitudes/  practice | Whether women received pre-test HIV counselling, took a HIV test and obtained result, received post-test HIV counselling during the antenatal period | Women with disabilities were less likely to receive pre-test HIV counselling, obtain HIV test results, and were less likely to receive post-test HIV counselling and all HTC services. |
| Chipanta et al. 2022 | Zambia | Women 16-55 years | Cross-sectional survey | WG Short Set | Participants were asked about difficulty in six functional domains: seeing, hearing, walking, cognition, self-care, and communication. Responses were scored across all domains as no difficulty (0), a little (2), a lot (3), and cannot do at all (4). Disability was defined as mild (2-5), moderate (6-8) and severe (9-24). Disability type was defined as a response of ‘a lot of difficulty’ or ‘cannot do at all’ in the individual domain. | Knowledge/  attitudes/  practice | Having tested for HIV during an antenatal care visit | Associations varied by disability type and severity. Women with moderate disability and mild disability, as well as those with cognitive disability reported more testing for HIV than women without disability. Women with hearing impairment were found to report less HIV testing than women without disability. |
| **Multiple regions** | | | | | | | | |
| Rotenberg et al. 2024b | Algeria, Argentina, Bangladesh, Central African Republic, Chad, Costa Rica, Cuba, Dominican Republic, Democratic Republic of Congo, The Gambia, Ghana, Guinea Bissau, Honduras, Iraq, Kiribati, Lao, Lesotho, Madagascar, Malawi, Mongolia, Nepal, Pakistan, Palestine, Samoa, Sao Tome and Principe, Sierra Leone, Suriname, Togo, Tunisia, Zimbabwe | 2-4 years | Cross-sectional survey | WG/UNICEF CFM | Caregivers were asked about whether the child experienced difficulty in eight functional domains: vision, hearing, communication, walking, controlling behaviour, learning, fine motor skills and playing. Disability was defined as a response of 'a lot of difficulty' or 'cannot do at all' in at least one of domains. | Morbidity | Measures of malnutrition (stunting, wasting, underweight) | Children with disability were found to be significantly more likely to experience all forms of malnutrition, including stunting, wasting and being underweight, than children without disability. |
| Marella et al. 2014 | Bangladesh and Fiji | ≥18 years | Cross-sectional survey | Rapid Assessment of Disability survey | Participants were asked about difficulty in eight functional domains: vision, hearing, communication, mobility, gross and fine motor, cognitive, appearance, and psychological distress. Disability was defined as having difficulty 'most' or 'all of the time' even with the use of assistive devices in at least one item from the first seven domains, or at least two items from the psychological distress domain. | Access; Other: Well-being | Well-being, access to the community | In Bangladesh and Fiji people with disability were found to have significantly worse well-being scores than people without disability. People with disability were also found to have worse access to all community sectors except legal assistance, drinking water and toilets. |
| Mactaggart et al. 2016 | Cameroon and India | ≥5 years | Nested case-control study | WG Extended Set; WG/UNICEF CFM | Caregivers of children aged 5-8 years, children aged 9-17 years, and adults aged 18 years or older reported difficulties in basic and complex activity domains. Disability was defined as a participants or proxy providing a response of 'a lot' or 'cannot do at all' in at least one 'basic' domain. | Knowledge/  attitudes/  practice; Morbidity | Serious health problems, chronic health conditions (high blood pressure, diabetes, arthritis, heart disease), health care seeking behaviour (for serious health problems, antenatal care, vaccination of children) | In both Cameroon and India people with disability had higher odds of serious health problems in the last year compared to people without disability. Health care seeking was found to be similar between people with disability and people without disability. Knowledge of rehabilitations services and access to these services was also found to be low in both countries for people with disability. |
| Prynn et al. 2021 | Cameroon, Guatemala, Haiti, India, Nepal and the Maldives | ≥60 years | Nested case-control study | WG Extended Set (Cameroon, Guatemala, India, the Maldives and Nepal); WG Short Set (Haiti) | Participants were asked about difficulty in functional domains seeing, hearing, walking, cognition, self-care, and communication (all countries) and anxiety or depression (Cameroon, Guatemala, India, the Maldives and Nepal only). Disability was defined as a response of 'a lot of difficulty' or 'cannot do at all' in one or more domains (all countries); or 'a lot' of anxiety/depression 'daily' (Cameroon, Guatemala, India, the Maldives and Nepal only). | Access; Knowledge/  attitudes/  practice; Morbidity | Catastrophic health expenditure, hypertension, diabetes, quality of life | In Guatemala older adults with disability were more likely to report serious health problems in the last 12 months and were four times more likely to report difficulty understanding information provided to them at health facilities than older adults without disability. In Guatemala and the Maldives older adults with disability were more likely to be diagnosed with diabetes than older adults without disability. In Nepal and the Maldives household healthcare expenditure was higher in households containing older adults with disability than households without. |
| Rotenberg et al. 2024a | Fiji, Kiribati, Mongolia, Samoa, Tonga, Tuvalu, Belarus, Georgia, Kosovo, Kyrgyzstan, Montenegro, Turkmenistan, Uzbekistan, Costa Rica, Cuba, Dominican Republic, Guyana, Honduras, Suriname, Algeria, Iraq, Palestine, Tunisia, Bangladesh, Nepal, Central African Republic, Chad, Democratic Republic of Congo, Gambia, Ghana, Guinea Bissau, Madagascar, Malawi, Sao Tome and Principe, Sierra Leone, Togo, Zimbabwe | 15-49 years | Cross-sectional survey | WG Short Set; WG/UNICEF CFM | Participants were asked about difficulty in functional domains: seeing, hearing, walking, remembering/concentrating, self-care, communication (all ages); making friends, controlling behaviour, accepting change, learning, communication, anxiety and depression (15-17 years). Disability was defined as a response of 'a lot of difficulty' or 'cannot do at all' in at least one domain (all ages), or ‘daily’ for domains or anxiety and depression (15-17 years). | Knowledge/  attitudes/  practice | Knowledge about HIV prevention, knowledge about mother-to-child-transmission, knowledge of a place to be tested for HIV, having ever tested for HIV, having tested for HIV in the last 12 months, knowing the HIV test results | People with disability were less likely to have comprehensive knowledge of HIV prevention and places to be tested for HIV than people without disability. Women with disability were also less likely to know how to prevent mother-to-child-transmission of HIV and to have ever been tested for HIV than women without disability. |
| Wallace et al. 2020 | Guatemala, Maldives, Nepal, India and Cameroon | ≥18 years | Cross-sectional survey | WG Extended Set^b^ | The WG Extended Set was used to measure functional disability in three domains: seeing, hearing and mobility. Disability was defined as a response of 'a lot of difficulty' or 'cannot do at all' in at least one of the three domains. The WG Extended Set anxiety and depression domains were also measured in this study but were used to define outcomes not the exposure. | Morbidity | Anxiety and depression | In all of the included countries adults with mobility, vision and functional difficulties were found to have increased odds of severe depression and severe anxiety. Hearing impairment was associated with increased odds of severe depression and severe anxiety in Guatemala and Nepal only. For all disability types and in all country settings functional difficulties were found to be more strongly associated with severe depression and anxiety than moderate depression and anxiety. |

ADL – Activities of daily living; AIDS - Acquired immunodeficiency syndrome; ANC – Antenatal care; CFM – Child Functioning Module; HIV – Human immunodeficiency virus; IADL – Instrumental activities of daily living; ICF - International Classification of Functioning, Disability and Health; OPD – Organisation of Persons with Disabilities; UNICEF – United Nations Children’s Fund; WG – Washington Group; WHO – World Health Organization

^a^ Tool not specified; ^b^Used a modified version of tool

# **Table S3. Prevalence of disability found in included studies organised by study region**

| **First Author, Year** | **Country/ies** | **Study cohort** | **Disability prevalence as reported by each study** |
| --- | --- | --- | --- |
| **East Asia and the Pacific** | | | |
| He et al. 2019 | China | ≥45 years | ADL disability in one area 8.3%, ADL disability in two or more areas 8.2% |
| Shi et al. 2024 | China | ≥60 years | Overall 70.7% |
| Yan et al. 2023 | China | ≥60 years | 2015 54.4%, 2018 50.7% |
| Marella et al. 2016 | Philippines | ≥18 years | Quezon City 7.2%, Ligao City 14.0% |
| Wilbur et al. 2021 | Vanuatu | ≥5 years | Not reported |
| **Latin America and the Caribbean** | | | |
| Grushka et al. 2020 | Argentina | ≤99 years | Not reported |
| Barreto et al. 2023 | Brazil | ≥15 years | Overall 7.6% |
| Macarevich Condessa et al. 2021 | Brazil | ≥18 years | 9.2% |
| Silva et al. 2017 | Brazil | ≥60 years | Functional limitation 30.1% |
| Lopez-Gil et al. 2021 | Colombia | 13-17 years | Vision impairment 46.7%, Hearing impairment 25.6% |
| Kuper et al. 2018 | Guatemala | ≥2 years | Not reported |
| Casebolt 2024 | Haiti | Women 15-49 years | Mild disability 15.9%, Moderate disability 3.2%, More severe disability 0.8% |
| Danquah et al. 2015 | Haiti | ≥5 years | Overall 4.1% |
| de Castro et al. 2017 | Mexico | 2-17 years | 8.0% |
| Flores-Flores et al. 2018 | Peru | 65-80 years | 17.3% |
| Hernandez-Vasquez et al. 2023 | Peru | Migrants and refugees from Venezuela, ≥18 years | 2.1% |
| Rohrer et al. 2010 | Peru | ≥18 years | 41.1% |
| **Middle East and North Africa** | | | |
| Trani et al. 2018 | Morocco and Tunisia | Whole population | Not reported |
| **South Asia** | | | |
| Casebolt et al. 2022 | India | Women 15-49 years | 0.9% |
| Casebolt et al. 2023 | India | Women 15-49 years | 1.2% |
| Grills et al. 2017 | India | ≥18 years | Overall 6.8% |
| Gudlavalleti et al. 2014 | India | ≥18 years | Not reported |
| Mathias et al. 2018 | India | ≥18 years | Psychosocial disability 4.8% |
| Muhammad et al. 2022 | India | ≥60 years | ADL disability: 7.4%, IADL disability 56.6%, Vision impairment 59.1%, Cognitive impairment 60% |
| Murthy et al. 2014 | India | Women 15-45 years | Not reported |
| Devkota et al. 2017 | Nepal | Women 15-49 years | Not reported |
| Devkota et al. 2021 | Nepal | Women 15-49 years | Not reported |
| Hameed et al. 2023 | Pakistan | Women 15-49 years | At risk of disability 11.5%, Living with disability 2.6% |
| Mahmood et al. 2022 | Pakistan | Women 15-49 years | Disability 14.1%, Severe disability 2.6% |
| Banks et al. 2020 | The Maldives | ≥2 years | Population based survey: 6.8% |
| Banks et al. 2022 | The Maldives | ≥2 years | Not reported |
| **Sub-Saharan Africa** | | | |
| Onadja et al. 2013 | Burkina Faso | ≥15 years | One functional limitation: Males 15.1%, Females 16.8%, Two or more functional limitations: Males 8.4%, Females 15.7% |
| DeBeaudrap et al. 2019 | Cameroon | 15-49 years | Not reported |
| Rotenberg et al. 2023 | Central African Republic, Chad, Democratic Republic of Congo, Gambia, Ghana, Lesotho, Madagascar, Malawi, Sierra Leone, and Togo | 2-4 years | Overall 7.0% |
| Massetti et al. 2024 | Lesotho | 13-24 years | Functional disability: Males 7.3%, Females 14.1% |
| Carew et al. 2019 | Liberia | Whole population | Not reported |
| Devendra et al. 2013 | Malawi | 2-9 years | Not reported |
| Prynn et al. 2020 | Malawi | ≥18 years | 7.6% |
| Aderemi et al. 2013 | Nigeria | 12-19 years | Not reported |
| Ekman et al. 2024 | Sierra Leone | 2-4 years | Functional difficulty 7.0%, Severe functional difficulty 2.0% |
| Trani et al. 2011 | Sierra Leone | ≥18 years | Not reported |
| Moodley et al. 2015 | South Africa | ≥18 years | 18.0% |
| Mutwali et al. 2019 | South Africa | ≥18 years | 11.0% |
| Myezwa et al. 2016 | South Africa | ≥18 years | Overall 45.7% |
| Pengpid et al. 2019 | South Africa | ≥15 years | 5.3% |
| Vergunst et al. 2017 | South Africa | ≥5 years | Not reported |
| Vergunst et al. 2019 | South Africa | ≥5 years | Not reported |
| Eide et al. 2015 | Sudan, Namibia, Malawi, and South Africa | Whole population | Not reported |
| Chipanta et al. 2023 | Tanzania | ≥15 years | Overall 9.9% |
| Abimanyi-Ochom et al. 2017 | Uganda | Women 15-49 years, men 15-54 years | Overall 16.6%, Single 11.5%, Multiple 5.1%, Low severity 15.4%, High severity 2.5%, Hearing disability 3.6% |
| Kwagala et al. 2021 | Uganda | Women 15-49 years | 4.0% |
| Zandam et al. 2021 | Uganda | Women 15-49 years | 15.9% |
| Chipanta et al. 2022 | Zambia | Women 16-55 years | Overall 29.9% |
| **Multiple regions** | | | |
| Rotenberg et al. 2024b | Algeria, Argentina, Bangladesh, Central African Republic, Chad, Costa Rica, Cuba, Dominican Republic, Democratic Republic of Congo, The Gambia, Ghana, Guinea Bissau, Honduras, Iraq, Kiribati, Lao, Lesotho, Madagascar, Malawi, Mongolia, Nepal, Pakistan, Palestine, Samoa, Sao Tome and Principe, Sierra Leone, Suriname, Togo, Tunisia, Zimbabwe | 2-4 years | Overall 6.6% |
| Marella et al. 2014 | Bangladesh and Fiji | ≥18 years | Bangladesh 10.5%, Fiji not reported |
| Mactaggart et al. 2016 | Cameroon and India | ≥5 years | Not reported |
| Prynn et al. 2021 | Cameroon, Guatemala, Haiti, India, Nepal and the Maldives | ≥18 years | Overall (from population-based survey): Cameroon 24.9%, Guatemala 22.4%, India 39.2%, the Maldives 29.4%, Nepal 9.7% (note Haiti not provided as used a different version of the WG questions) |
| Rotenberg et al. 2024a | Fiji, Kiribati, Mongolia, Samoa, Tonga, Tuvalu, Belarus, Georgia, Kosovo, Kyrgyzstan, Montenegro, Turkmenistan, Uzbekistan, Costa Rica, Cuba, Dominican Republic, Guyana, Honduras, Suriname, Algeria, Iraq, Palestine, Tunisia, Bangladesh, Nepal, Central African Republic, Chad, Democratic Republic of Congo, Gambia, Ghana, Guinea Bissau, Madagascar, Malawi, Sao Tome and Principe, Sierra Leone, Togo, Zimbabwe | 15-49 years | Total sample 4.8% |
| Wallace et al. 2020 | Guatemala, Maldives, Nepal, India and Cameroon | ≥18 years | Hearing impairment 1.0% - 3.7%, Vision impairment 0.7%-3.6%, Mobility impairment 2.0%-5.5% |

ADL – Activities of daily living; IADL – Instrumental activities of daily living; WG – Washington Group
